# Evaluating daily profile of central aortic pressure and reflected pulse wave parameters in climacteric women

**DOI:** 10.1186/s12872-021-02064-9

**Published:** 2021-05-19

**Authors:** Sergey N. Tolstov, Igor A. Salov, Anton R. Kiselev, Andrey P. Rebrov

**Affiliations:** 1 Department of Therapy with Courses in Cardiology, Functional Diagnostic and Geriatrics, Saratov State Medical University, Saratov, Russia; 2 Department of Obstetrics and Gynecology, Saratov State Medical University, Saratov, Russia; 3 Department of Innovative Cardiological Information Technology, Institute of Cardiological Research, Saratov State Medical University, Saratov, Russia; 4 Coordinating Center for Fundamental Research, National Medical Research Center for Therapy and Preventive Medicine, Moscow, Russia; 5 Department of Hospital Therapy, Saratov State Medical University, Saratov, Russia

**Keywords:** Menopause, Arterial stiffness, Central aortic pressure, Augmentation index, Pulse pressure amplification

## Abstract

**Introduction:**

Structural and functional changes of the vascular wall in women occur already at the very early stages of reproductive aging. An emergence of applanation tonometry made it possible to evaluate arterial stiffness and central hemodynamic parameters non-invasively, which considerably expanded the information that had been provided previously by invasive methods used for studying these parameters during cardiac catheterization. Whereas a few studies have assessed central aortic pressure (CAP) parameters and reflected pulse wave in women at different phases of their reproductive aging, none investigated the daily profile of CAP and reflected pulse wave parameters in women undergoing different stages of the menopause. Background: assessment of the daily variability in CAP and daily profile of amplification and augmentation of pulse blood pressure (PBP) in women at different menopause phases.

**Methods:**

The study involved 384 climacteric women. The first group included 168 women undergoing perimenopause, the second group comprised of 216 women in their early postmenopausal stage. A 24-h blood pressure (BP) monitoring in the brachial artery and aorta (BPLab® Vasotens® system, Petr Telegin LLC, Russia) was performed via the measurements of the following indicators: systolic blood pressure (SBP), pulse blood pressure (PBP), central aortic systolic pressure (CASP), central aortic pulse pressure (CAPP), aortic augmentation index (AIxao), and pulse pressure amplification (PPA).

**Results:**

When investigating PPA values in the brachial artery and aorta, we detected smaller amplification and higher aortic augmentation index at night than in daytime, which reflected a disproportionately higher CAP level during night hours. This pattern was more pronounced in postmenopausal women. We calculated the logistic regression equation (adjusted R^2^ = 0.49, log-likelihood = − 50.3, chi-square (19) = 97.6, *p* < 0.001), in which dependent variable was represented by the menopausal status, whereas body mass index with all indicators of a 24-h BP monitoring represented independent variables. In this model, two indicators (body mass index and AIxao) were, independently of each other, associated significantly with the menopause phases. Differences among women at various climacteric phases in terms of remaining indicators of a 24-h BP monitoring, apparently, matched the differences in their body mass index values.

**Conclusion:**

Rising CAP, in combination with declining PPA and augmenting reflected pulse wave amplitude, may be associated with an increased risk of cardiovascular complications.

## Introduction

Along with shared risk factors for cardiovascular disease (CVD) in men and women, women have a unique risk factor: the development of estrogen deficiency in postmenopausal period [1, 2]. Available contemporary data on the mechanisms of estrogenic influence on cardiovascular system indicate the complexity and multidirectional nature of their biological effects, provided by their impact on metabolic risk factors [3, 4], along with other direct impacts on the vascular wall [5–7]. Estrogens cause vasodilation by affecting the synthesis of nitric oxide (NO) and prostacyclin, reducing synthesis of endothelin-1, and promoting the opening of calcium channels in the cell membranes of vascular smooth muscle cells [8].

The effect of estrogen deficiency on CVD development can be mediated by various mechanisms. On one hand, age-related estrogen deficiency promotes the redistribution of adipose tissue resulting in abdominal obesity, development of insulin resistance, and occurrence of adverse metabolic changes, all of which create the basis for the formation of menopausal metabolic syndrome [9, 10].

On the other hand, the consequence of estrogen deficiency is an imbalance of vasoactive mediators with a predominance of vasoconstrictor production and a decrease in the secretion of vasodilators. As a result, the conditions are created for the development of endothelial dysfunction and changes in elastic properties of the vascular wall [7, 11].

Structural and functional changes of the vascular wall in women occur already at the very early stages of reproductive aging. They accelerate significantly after the onset of menopause, which is largely due to the loss of protective effect of female sex hormones on the cardiovascular system [11–13].

In most studies, the menopause onset has been associated with an imbalance among the production of vasoconstrictor and vasodilating factors: a decrease in endothelium-dependent vasodilation, related to insufficient NO production [14], and increase in vasoconstrictor factors [5, 11, 15].

Currently, non-invasive methods for assessing early structural and functional changes in the vascular wall are actively studied. Such methods include ultrasound examination of the carotid arteries, evaluation of endothelial function and coronary calcification, characterization of the central pulse wave, and investigation of the vascular wall for arterial stiffness. All of the above belong to subclinical vascular CVD markers, enabling the assessment of the vascular wall condition at different stages of CVD continuum [16, 17].

An emergence of applanation tonometry made it possible to evaluate arterial stiffness and central hemodynamic parameters non-invasively, which considerably expanded the information that had been provided previously by invasive methods used for studying these parameters during cardiac catheterization. The development of techniques, combining the brachial artery blood pressure (BP) measurements with a cuff and the analysis of the central pulse wave, permitted to evaluate the functional parameters of the arterial wall via the method of outpatient daily monitoring [16, 17], which expanded the possibilities of early cardiovascular screening of the patients at risk for CVD.

The phenomenon of pulse pressure amplification (PPA) from the central arteries to the peripheral arteries, mainly because of a rise in systolic pressure, makes it incorrect to use pulse pressure in the brachial artery as a substitute for pulse pressure in aorta.

Taking into account that central aortic pressure (CAP) reflects blood flow in the coronary and cerebral vasculatures to a greater extent and is a more significant predictor of cardiovascular complications, the assessment of central pulse wave parameters has additional advantages for characterizing the state of the cardiovascular system than assessment of arterial stiffness indicators alone [17].

Whereas a few studies have assessed CAP parameters and reflected pulse wave in women at different phases of their reproductive aging [18–20], none investigated the daily profile of CAP and reflected pulse wave parameters in women undergoing different stages of the menopause. Our preliminary results established an association of menopause phases with some hemodynamic parameters (CAP parameters, vascular stiffness, and reflected pulse wave) [21]. Nonetheless, an impact of some cardiovascular risk factors (e.g., hypertension and obesity), as well as complex structure of revealed association deserves further comprehensive investigation.

The objective of our study was to assess daily variability in CAP and daily profile of amplification and augmentation of pulse blood pressure (PBP) in women at different menopause phases, with regards to the presence, or absence, of some cardiovascular risk factors, such as hypertension and obesity.

## Materials and methods

### Patients

The study included 384 women with a median age of 51 years old (lower and upper quartiles: 48, 56) with menstrual dysfunction and manifestations of menopausal syndrome of varying severity and duration. According to the criteria for the aging of the female reproductive system—STRAW + 10, study subjects were assigned to the late perimenopausal and early postmenopausal stages [22]. For patients with hypertension, an additional inclusion criterion was the absence of regular antihypertensive therapy during the last three months before enrollment in the study. All subjects underwent a complete clinical examination prior to their enrollment. It should be also noted that among 189 hypertensive patients included in the study, 77 subjects (40.7%) had hypertension diagnosed for the first time during the preliminary clinical examination.

The non-inclusion criteria for our study were as follows: the presence of a clinically manifested atherosclerosis; type 1 and type 2 diabetes mellitus; the development of reproductive arterial hypertension and symptomatic arterial hypertension; cardiac arrhythmias; thyroid disease; premature or early menopause; hysterectomy; ovariectomy; severe somatic disorders.

After obtaining written consent to participate in the study, all examined patients were split among two groups based on their menopausal status: Group 1 consisted of 168 women undergoing perimenopause, while Group 2 included 216 women in their early postmenopausal stage.

Major clinical characteristics of examined women are presented in Table 1. The age of study participants in Group 1 was 49 years old (48, 50); whereas in Group 2, it was 53 years old (50, 55). Women in the early postmenopausal period (Group 2) exhibited an increase in the frequency of occurrence of major risk factors for CVD (hypertension, lipid metabolism disorders, overweight condition and obesity, and metabolic syndrome), as well as in their severity, compared with women undergoing menopausal transition (Group 1). Such trends were quite expected, given the difference in their menopausal status, underlying the assignment of patients into the study groups, since the menopause onset is thought to be associated with an increase in the frequency and severity of major cardiovascular risk factors [1, 2].

**Table 1:** Clinical characteristics of women included in the study

Indicators	Group 1 (n = 168)	Group 2 (n = 216)	*p*_1-2_
Age of patients, years	48.7(48.0, 50.0)	53.0 (50.0, 55.0)	< 0.001
Menopause duration, years	–	3.5 (2.0, 5.0)	–
Hypertension, n (%)	72 (38.1)	117/61.9	0.020
BMI, kg/m^2^	25.6(24.0, 26.5)	28.6(25.9, 33.2)	< 0.001
BMI > 25 kg/m^2^, n (%)	87 (51.8%)	170 (78.7%)	< 0.001
WC, cm	87.0(74.0, 94.0)	94.0(87.0, 108.0)	< 0.001
WC > 80 cm, n (%)	84 (50%)	191 (88.4%)	< 0.001
WC/TC	0.81 (0.77, 0.86)	0.86 (0.82, 0.92)	< 0.001
Metabolic syndrome, n (%)	39/23.2	116/54	< 0.001
*Antihypertensive therapy regimen for women with hypertension:*	(n = 72)	(n = 117)	
Losartan (100 mg per day), n (%)	68 (94.4%)	39 (33.3%)	< 0.001
Losartan (100 mg per day) + indapamide (1.5 mg per day), n (%)	4 (5.6%)	64 (54.8%)	< 0.001
Losartan (100 mg per day) + indapamide (1.5 mg per day) + amlodipine (5 mg per day), n (%)	0 (0%)	14 (11.9%)	0.057

The quantitative data are presented as Me (LQ, UQ); the qualitative indicators are in the form of absolute and relative frequencies; *p*_1-2_ represents the significance of differences between the women of Groups 1 and 2; BMI is a body mass index; WC stands for waist circumference; WC/TC is the ratio of the waist circumference to the circumference of thighs

Antihypertensive therapy in all examined women with arterial hypertension was conducted with a medication from the group of angiotensin II receptor antagonists—losartan, with a gradual dose titration up to 100 mg per day. Whenever it was necessary to prescribe a combination therapy, a sustained-release formulation of indapamide was added at a fixed dose of 1.5 mg per day: in 4 (5.6%) subjects of Group 1 and in 64 (54.8%) patients of Group 2. In Group 2, 14 (11.9%) women required triple antihypertensive therapy with the addition of 5 mg of amlodipine per day (Table 1).

Taking into account the heterogeneity of our experimental groups, along with the possibility of impact by some cardiovascular risk factors on the studied parameter values, we additionally investigated the daily changes in the amplification and augmentation of pulse pressure in healthy women of the climacteric period. Hence, we identified separate subgroups of women without arterial hypertension, overweight condition or obesity at the time of inclusion in the study from previously formed groups of women undergoing menopausal transition (Group 1a, n = 78) and early postmenopausal period (Group 2a, n = 41). The subgroups in question were comparable in all clinical characteristics, except for age (46.4 ± 4.9 years in the subgroup 1a vs. 51.7 ± 5.6 years in the subgroup 2a, *p* < 0.001), which was expected, given their different menopausal statuses.

### Data recording

A 24-h monitoring of blood pressure and the study of arterial stiffness were performed by the oscillometric method using the MnSDP-2 apparatus and the BPLab® software in the expanded Vasotens® edition.

The BPLab® Vasotens® system (Petr Telegin LLC, Russia; URL: http://vasotens.com/) registers a pulse wave on the brachial artery and, possessing a transforming function, allows obtaining additional information on CAP and vascular wall stiffness. There are publications on successful validation of this device and its use for general population [23–26].

We analyzed daily averages of systolic blood pressure (SBP) and pulse blood pressure (PBP). The average shape of pulsations in the ascending aorta was used to calculate the CAP parameters: central aortic systolic pressure (CASP), central aortic pulse pressure (CAPP), aortic augmentation index (AIxao) and pulse pressure amplification [16]. Pulse pressure amplification (PPA, mmHg) was estimated by the difference between PBP и CAPP: PPA = PBP – CAPP.

### Statistical analysis

Statistical data processing was performed using the STATISTICA 10.0 software package (StatSoft, USA; URL: http://statsoft.ru/products/STATISTICA_Base/). For quantitative parameters, in case of a normal distribution, the studied characteristics were presented as means with their standard deviations, M ± SD. Whenever the studied traits did not comply with a normal distribution, they were presented as medians with lower and upper quartiles, Me (LQ, UQ). We investigated how well the distribution types of the measured variables matched to the law of normal distribution using the Shapiro–Wilk test. The following variables exhibited a normal distribution: SBP, PBP, CASP, CAPP, PPA, and AIxao (in subgroups 1a and 2a). All quantitative clinical parameters (age, menopause duration, body mass index, waist circumference, the ratio of the waist circumference to the circumference of thighs), and AIxao (in Groups 1 and 2) were non-normally distributed. For qualitative indicators, absolute and relative frequencies were calculated.

For quantitative indicators, differences between the groups were analyzed using the unequal variance t-test in case of a normal distribution (note: on most occasions, the Levene’s test yielded unequal variances for the traits among the compared groups), otherwise we employed non-parametric Mann–Whitney criterion. Multiple logistic regression analysis was used to assess multiple relationships between the parameters. The significance level in our study was set at *p* < 0.05.

## Results

We compared the absolute values of the SBP, PBP, CASP, CAPP and the PPA phenomenon among women during their perimenopause (Group 1) and early postmenopause (Group 2), separately for daytime and nighttime hours. A comparison of the daily BP profile indicators in the brachial artery *vs*. aorta with simultaneous non-invasive 24-h BP monitoring in women undergoing different phases of the menopause is presented in Table 2.

**Table 2:** Indicators of a 24-h BP monitoring in the brachial artery and aorta in women at various climacteric phases

Indicators	Group 1 (n = 168)	Group 2 (n = 216)	*p*_1-2_
SBP—24-h period, mmHg	115.7 ± 11.3	126.2 ± 13.3	< 0.001
SBP—day, mmHg	118.5 ± 11.8	130.7 ± 13.9	< 0.001
SBP—night, mmHg	106.5 ± 12.1	116.8 ± 14.9	0.001
PBP—24-h period, mmHg	43.7 ± 6.2	49.3 ± 9.5	0.014
PBP—day, mmHg	45.6 ± 6.5	52.0 ± 10.0	0.032
PBP—night, mmHg	40.9 ± 6.2	47.4 ± 9.6	0.013
CASP—24-h period, mmHg	108.4 ± 10.8	118.2 ± 12.0	< 0.001
CASP—day, mmHg	109.7 ± 10.7	119.6 ± 12.3	0.001
CASP—night, mmHg	99.3 ± 10.1	110.5 ± 15.6	0.012
CAPP—24-h period, mmHg	34.1 ± 5.8	39.9 ± 8.0	< 0.001
CAPP—day, mmHg	34.4 ± 6.0	40.6 ± 8.4	0.002
CAPP—night, mmHg	32.3 ± 5.4	39.4 ± 11.9	0.001
PPA—24-h period, mmHg	9.6 ± 2.1	9.5 ± 2.2	0.326
PPA—day, mmHg	11.2 ± 2.2	11.4 ± 2.2	0.188
PPA—night, mmHg	8.6 ± 1.9**	8.0 ± 1.7**	0.002
AIxao—24-h period, %	22 (15–28)	28 (20–35)	0.004
AIxao—day, %	20 (12, 25)	27.5 (22, 35)	0.018
AIxao—night, %	23.5 (17, 28)*	32 (22, 38)*	0.001

The data are presented in the form of M ± SD or Me (LQ, UQ); *p*_1-2_ represents the significance of differences between the women of Groups 1 and 2; asterisks show the significance level of the differences between the daytime and nighttime monitoring periods (*p < 0.05; **p < 0.01); SBP stands for systolic blood pressure; PBP indicates pulse blood pressure; CASP is central aortic systolic pressure; CAPP is central aortic pulse pressure; AIxao is aortic augmentation index; PPA represents pulse pressure amplification

The dynamics of SBP and PBP during the 24-h period in women of both groups matched the changes in similar indicators measured in the brachial artery. For postmenopausal women, we discovered higher values of SBP and PBP both in the brachial artery and aorta during the 24-h period, in daytime hours (when staying awake) and at night (during sleep), compared with women undergoing menopausal transition.

For women of both groups, our results demonstrated that CAPP at night declined less than peripheral PBP; while in women of Group 2, the decrease of CAPP at night was less pronounced than in Group 1. The reduction in CAPP at night was 2.1 ± 1.1 mmHg in Group 1 and 1.2 ± 1.0 mmHg in Group 2 (*p* = 0.001). The decline in peripheral PBP at night in women of Group 1 was 4.7 ± 1.4 mmHg, while in women of Group 2, it was 4.6 ± 1.2 mmHg (*p* = 0.452).

With similar changes in the 24-h BP profile in women of both groups, a smaller decline in central PBP at night may indicate a greater CASP augmentation during this time of the day.

In connection with revealed disproportionality in the degree of a night-time decline in peripheral and central BP, we analyzed AIxao (Table 2). To diminish the dependence of AIxao on the heart rate, the normalized value of the former, corresponding to the heart rate of 75 beats per minute, was calculated. Thus, we leveled off the effect of different heart rate values on the studied parameter in daytime and nighttime hours.

In women of the early postmenopausal stage, compared with those in perimenopause, we detected lower PPA values over 24-h period, at night, and a clear trend of its decrease in daytime hours.

In subjects of both groups, PPA values at night were lower than daytime values, although a more pronounced difference was noted in women of the early postmenopausal stage. These changes were associated with higher values of AIxao during 24-h period, daytime and nighttime in women at early postmenopausal phase *versus* the women undergoing perimenopause.

Analyzing 24-h AIxao dynamics, we noticed its higher nighttime values, compared with daytime monitoring period, with more pronounced changes detected in women of the early postmenopausal stage.

Twenty-four-hour changes in amplification and augmentation of pulse pressure in subgroups 1a and 2a are presented in Table 3.

**Table 3:** Indicators of a 24-h BP monitoring in the brachial artery and aorta in healthy women at various climacteric phases (subgroups 1a and 2a)

Indicators	Subgroup 1a, n = 78	Subgroup 2a, n = 41	*p*_1a-2a_
SBP—24-h period, mmHg	111.3 ± 11.8	116.2 ± 12.1	0.460
SBP—day, mmHg	114.5 ± 12.1	118.5 ± 12.9	0.096
SBP—night, mmHg	104.8 ± 10.5	109.4 ± 12.8	0.037
PBP—24-h period, mmHg	41.2 ± 7.4	43.6 ± 9.2	0.125
PBP—day, mmHg	43.8 ± 7.6	48.4 ± 8.3	0.029
PBP—night, mmHg	38.4 ± 7.2	42.8 ± 8.5	0.018
CASP—24-h period, mmHg	103.5 ± 10.2	106.8 ± 10.9	0.104
CASP—day, mmHg	106.4 ± 10.9	112.2 ± 11.5	0.007
CASP—night, mmHg	98.5 ± 9.3	102.6 ± 9.8	0.026
CAPP—24-h period, mmHg	32.1 ± 6.1	34.7 ± 6.2	0.030
CAPP—day, mmHg	32.4 ± 5.8	36.7 ± 6.6	0.001
CAPP—night, mmHg	29.6 ± 5.6	34.3 ± 5.9	0.001
PPA—24-h period, mmHg	9.2 ± 4.3	9.0 ± 2.9	0.394
PPA—day, mmHg	11.4 ± 5.8	11.8 ± 3.2	0.443
PPA—night, mmHg	8.8 ± 1.9**	8.5 ± 2.1**	0.411
AIxao—24-h period, %	21.8 ± 11.3	26.2 ± 11.8	0.049
AIxao—day, %	19.5 ± 9.2	24.5 ± 10.8	0.014
AIxao—night, %	23.0 ± 10.6*	28.2 ± 11.2*	0.034

The data are presented in the form of M ± SD; *p*_1a-2a_ represents the significance of differences between the women of subgroups 1a and 2a; asterisks show the significance level of the differences between the daytime and nighttime monitoring periods (*p < 0.05; **p < 0.01); CAPP stands for central aortic pulse pressure; AIxao is aortic augmentation index; PBP indicates pulse blood pressure in the brachial artery; PPA is pulse pressure amplification

The discovered dynamics in the 24-h PPA profiles of healthy women (subgroups 1a and 2a) matched the identified changes in groups of climacteric women (Groups 1 and 2): the CAPP at night declined less than the peripheral BP, measured on the brachial artery, and the PPA at night was less pronounced than in daytime hours.

The decrease of peripheral PBP at night in women was 5.4 ± 1.6 mmHg in the subgroup 1a and 5.6 ± 1.6 mmHg in the subgroup 2a (*p* = 0.259). Despite the absence of significant differences in the degree of the CAPP decrease, women in subgroup 2a showed a clear tendency towards a smaller CAPP decline at night than subjects in the subgroup 1a (2.4 ± 1.2 vs. 2.8 ± 1.2 mmHg, *p* = 0.086).

The 24-h AIxao dynamics indicated its higher nighttime values ​​in both groups, whereas more pronounced changes were noted in postmenopausal women.

Thus, in women of early postmenopausal phase, compared with those undergoing menopausal transition, we detected a decrease in 24-h and nighttime PPA profiles, and a clear trend of PPA decline in daytime hours as well, combined with higher augmentation index values ​​ during the day, night, and over a 24-h monitoring period. Analyses of 24-h PPA and AIxao profiles in women of both groups demonstrated PPA reduction accompanied by its higher values at night, compared with daytime hours, with more distinct changes noted in women of the early postmenopausal period.

We conducted multiple logistic regression analysis, in which the menopausal status was used for dependent variable. Body mass index and all indicators of a 24-h BP monitoring in the brachial artery and aorta in women of Groups 1 and 2 were included in the regression model as independent variables. The outcomes of logistic regression analysis are presented in Table 4. General characteristics of the calculated logistic regression equation included adjusted R^2^ = 0.49, log-likelihood = − 50.3, chi-square (19) = 97.6, *p* < 0.001. We conducted multiple regression analysis, in which the menopausal status was used for dependent variable. For each independent trait, a standardized value of the regression coefficient (beta) was calculated. The beta coefficients for various parameters were as follows: 0.652 (*p* = 0.007) for CAPP, 0.609 (*p* = 0.044) for AIxao, − 0.580 (*p* = 0.081) for PPA, 0.028 (*p* = 0.204) for CASP, 0.215 (*p* = 0.385) for SBP, and 0.205 (*p* = 0.326) for PBP. General characteristics of the obtained multiple regression equation included adjusted R^2^ = 0.80, F(13.69) = 26.9, *p* < 0.001.

**Table 4:** Relationship between the menopausal status, body mass index and indicators of a 24-h BP monitoring in the brachial artery and aorta in women: The results of logistic regression analysis

	OR	SE	95% CI	z	*p*	Coef	SE
BMI	1.82	0.29	1.32–2.50	3.69	< 0.001	0.60	0.16
SBP—24-h period	1.14	0.18	0.83–1.57	0.83	0.404	0.13	0.16
SBP—day	1.10	0.16	0.83–1.46	0.65	0.518	0.09	0.15
SBP—night	1.03	0.09	0.86–1.23	0.36	0.721	0.03	0.09
PBP—24 h period	2.53	1.44	0.83–7.70	1.63	0.103	0.93	0.57
PBP—day	0.90	0.46	0.33–2.47	− 0.21	0.837	− 0.11	0.52
PBP—night	0.61	0.23	0.29–1.27	− 1.33	0.185	− 0.50	0.37
CASP—24 h period	0.91	0.09	0.76–1.09	− 1.01	0.313	− 0.09	0.09
CASP—day	0.90	0.08	0.76–1.07	− 1.17	0.241	− 0.10	0.09
CASP—night	0.93	0.05	0.84–1.04	− 1.25	0.211	− 0.07	0.06
CAPP—24 h period	0.91	0.09	0.74–1.11	− 0.97	0.333	− 0.10	0.10
CAPP—day	0.45	0.21	0.18–1.13	− 1.71	0.087	− 0.81	0.47
CAPP—night	1.49	0.51	0.77–2.92	1.18	0.239	0.40	0.34
PPA—24 h period	0.44	0.22	0.16–1.19	− 1.62	0.106	− 0.82	0.51
PPA—day	1.24	0.41	0.65–2.36	0.65	0.516	0.21	0.33
PPA—night	1.21	0.27	0.78–1.88	0.84	0.402	0.19	0.22
AIxao—24 h period	0.28	0.14	0.10–0.77	− 2.46	0.014	− 1.29	0.52
AIxao—day	2.30	0.81	1.16–4.58	2.38	0.017	0.83	0.35
Aixao—night	1.51	0.26	1.08–2.13	2.40	0.016	0.42	0.17
_constant	4.96e + 16	8.24e + 17	362.96–6.78e + 30	2.31	0.021	38.44	16.61

OR, odds ratio; SE, standard errors; CI, confidential interval; Coef., coefficients; BMI, body mass index; SBP, systolic blood pressure; PBP, pulse blood pressure in the brachial artery; CASP, central aortic systolic pressure; CAPP, central aortic pulse pressure; PPA, pulse pressure amplification; AIxao, aortic augmentation index

## Discussion

The phenomenon of pulse pressure amplification from central to peripheral arteries implies that PBP use in brachial artery as a substitute for PBP in aorta is quite inadequate. It was previously shown that CAP (especially CAPP), along with augmentation index, correlate with the degree of transformation in large arteries and pulse wave velocity as a conventional indicator of the vascular wall stiffness [27, 28].

Considering important clinical and prognostic values of a 24-h peripheral BP profile, the data on comparability of indicators, recorded during a 24-h BP monitoring in the brachial artery *vs*. aorta, are of indisputable interest. Up to now, the problem of a 24-h variability of both CAP and parameters of reflected wave in a simultaneous non-invasive BP monitoring in the brachial artery and aorta in climacteric women was not sufficiently addressed, which led to this research.

Previously conducted studies have shown comparable circadian rhythms for both central blood pressure and peripheral blood pressure; while less pronounced decrease in central SBP was observed, compared with the peripheral SBP at night in menopausal women. However, the mechanisms explaining this phenomenon remain insufficiently studied [29].

In our study of climacteric women, we established lower PPA values at night than in daytime hours, while comparing PBP in the brachial artery and aorta, which reflected a disproportionately higher level of CAP at night *vs.* daytime hours. The 24-h AIxao profile was characterized by higher values at night, compared with the daytime period. This pattern was more pronounced in postmenopausal women.

It could be assumed that age-related estrogen deficiency, leading to structural and functional changes in the vascular wall, may affect the amplification and augmentation of pulse pressure.

The research conducted by Kuznetsova et al. [26] included women of an age range comparable to our study. However, the study carried out on women in this age range requires detailing their menopausal status (the period of the menopausal transition or early postmenopause), which was demonstrated by our results. Besides, the research of Kuznetsova et al. [26], in contrast to our study, involved solely healthy volunteers, thus excluding various additional risk factors, largely associated with the menopause.

It can be assumed that higher CAPP values, combined with a lower PPA and an amplitude increase of the reflected pulse wave, determine the pulse wave damaging effect on the blood vessels of the target organs and, therefore, may be associated with increased risk of cardiovascular complications, as was shown in a number of studies [28, 30].

We were interested in the effect of the menopause per se on the discovered phenomenon of disproportionality in augmentation and amplification of pulse pressure in menopausal women. The data in Tables 1 and 2 clearly imply that body mass index and all indicators of a 24-h BP monitoring in the brachial artery and aorta differed significantly among the groups of women at various climacteric phases. Therefore, the association between the body mass index and the studied indicators of a 24-h BP monitoring comes as no surprise. However, after the inclusion of the body mass index in the regression model, the association of climacteric phases with the aortic augmentation index persists throughout the 24-h cycle, whereas no significant relationship between the pulse pressure amplification and menopausal status was established (Table 4). The implications of this outcome are especially interesting, given that previous studies have shown a positive effect of the menopausal hormone therapy on amplification increase and pulse pressure augmentation decrease in postmenopausal women [31].

The value of the augmentation index could be potentially considered as a criterion for the magnitude of a cardiovascular risk. An increase in the amplitude of the reflected central pulse wave leads to a significant increase in the afterload, myocardial hypertrophy, augmented oxygen demand, and a decrease in the efficiency of subendocardial blood flow [30].

## Conclusion

In women at the late phase of the menopausal transition and early postmenopausal phase, a decrease in the differences between central and peripheral nocturnal BP was revealed (smaller amplification of peripheral BP at night *vs*. daytime), which was associated with a greater CASP augmentation during daytime hours. This pattern was more pronounced in women at the postmenopausal stage.

### Study limitations

An important limitation of our research is related to the heterogeneity of the studied groups of women in terms of major clinical characteristics, such as percentages of hypertensive subjects, patients with a body mass index exceeding 25 kg/m^2^, etc. Such variability is caused primarily by their menopausal status, making it virtually impossible to select clinically comparable groups without introducing additional systematic errors. We realize that these differences may affect the values ​​of blood pressure, augmentation index, and other studied parameters. However, we suggest to consider such unavoidable limitation in the context of the differences in menopausal status, underlying the assignment of the subjects in our study to different experimental groups.

Despite the simplicity and accessibility of technology for assessing 24-h monitoring of central hemodynamic parameters on outpatients, further studies are certainly necessary before such methods are firmly introduced into routine clinical practice.

The pulse wave analysis largely depends on the quality of recorded signals. Therefore, inaccuracies, accompanying outpatient measurements, may limit the overall evaluation precision. It should be taken into account that revealed differences in circadian fluctuations of PPA and augmentation in women of both groups may be related to their age (e.g., a regular PPA decrease with age), differences in the frequency of occurrence of arterial hypertension and metabolic syndrome, and with differences in their body position during daytime *versus* nighttime hours.

## Data Availability

The datasets generated and/or analyzed during the current study are not publicly available due the Policies for Access to Clinical Data of Saratov State Medical University but are available from the corresponding author upon reasonable request.

## Abbreviations

AIxao: Aortic augmentation index
BMI: Body mass index
BP: Blood pressure
CAP: Central aortic pressure
CAPP: Central aortic pulse pressure
CASP: Central aortic systolic pressure
CVD: Cardiovascular disease
LQ: Lower quartile
M: Mean
Me: Median
NO: Nitric oxide
PBP: Pulse blood pressure
PPA: Pulse pressure amplification
SBP: Systolic blood pressure
SD: Standard deviation
UQ: Upper quartile
WC: Waist circumference
WC/TC: The ratio of the waist circumference to the circumference of thighs

## References

[1] Newson L (2018). Menopause and cardiovascular disease. Post Reprod Health.

[2] Yang XP, Reckelhoff JF (2011). Estrogen, hormonal replacement therapy and cardiovascular disease. Curr Opin Nephrol Hypertens.

[3] Kolovou GD, Bilianou HG (2008). Influence of aging and menopause on lipids and lipoproteins in women. Angiology.

[4] Palmisano BT, Zhu L, Stafford JM (2017). Role of estrogens in the regulation of liver lipid metabolism. Adv Exp Med Biol.

[5] Mendelsohn M, Karas R (1999). The protective effects of estrogen on the cardiovascular system. N Engl J Med.

[6] Resanovic I, Rizzo M, Zafirovic S, Bjelogrlic P, Perovic M, Savic K (2013). Anti-atherogenic effects of 17β-estradiol. Horm Metab Res.

[7] Hou X, Pei F (2015). Estradiol inhibits cytokine-induced expression of VCAM-1 and ICAM-1 in cultured human endothelial cells via AMPK/PPARα activation. Cell Biochem Biophys.

[8] Moreau KL, Stauffer BL, Kohrt WM, Seals DR (2013). Essential role of estrogen for improvements in vascular endothelial function with endurance exercise in postmenopausal women. J Clin Endocrinol Metab.

[9] Spencer C, Goldsland I, Stevenson J (1997). Is there a menopausal metabolic syndrome?. Gynecol Endocrinol.

[10] Stefanska A, Bergmann K, Sypniewska G (2015). Metabolic syndrome and menopause: pathophysiology, clinical and diagnostic significance. Adv Clin Chem.

[11] Cid MC, Schnaper HW, Kleinman HK (2002). Estrogens and the vascular endothelium. Ann N Y Acad Sci.

[12] Lambrinoudaki I, Kazani A, Armeni E, Rizos D, Augoulea A, Kaparos G (2018). The metabolic syndrome is associated with carotid atherosclerosis and arterial stiffness in asymptomatic, nondiabetic postmenopausal women. Gynecol Endocrinol.

[13] Tsai SS, Lin YS, Hwang JS, Chu PH (2017). Vital roles of age and metabolic syndrome-associated risk factors in sex-specific arterial stiffness across nearly lifelong ages: possible implication of menopause and andropause. Atherosclerosis.

[14] Moreau KL, Hildreth KL, Meditz AL, Deane KD, Kohrt WM (2012). Endothelial function is impaired across the stages of the menopausetransition in healthy women. J Clin Endocrinol Metab.

[15] Klawitter J, Hildreth KL, Christians U, Kohrt WM, Moreau KL (2017). A Relative L-arginine deficiency contributes to endothelial dysfunction across the stages of the menopausal transition. Physiol Rep.

[16] Laurent S, Cockcroft J, Van Bortel L, Boutouyrie P, Giannattasio C, Hayoz D (2006). Expert consensus document on arterial stiffness: methodological issues and clinical applications. Eur Heart J.

[17] Vlachopoulos C, Xaplanteris P, Aboyans V, Brodmann M, Cífková R, Cosentino F, et al. The role of vascular biomarkers for primary and secondary prevention. A position paper from the European Society of Cardiology Working Group on peripheral circulation: Endorsed by the Association for Research into Arterial Structure and Physiology (ARTERY) Society. Atherosclerosis 2015;241:507–32. 10.1016/j.atherosclerosis.2015.05.007. 10.1016/j.atherosclerosis.2015.05.007 26117398

[18] Regnault V, Thomas F, Safar ME, Osborne-Pellegrin M, Khalil RA, Pannier B (2012). Sex difference in cardiovascular risk: role of pulse pressure amplification. J Am Coll Cardiol.

[19] Bordin Pelazza B, Filho SRF (2017). Comparison between central and brachial blood pressure in hypertensive elderly women and men. Int J Hypertens.

[20] Harvey RE, Johnson MC, Ranadive SM, Joyner MJ, Lahr BD, Miller VM (2017). Aortic hemodynamics in postmenopausal women following cessation of hormone therapy. Physiol Rep.

[21] Salov IA, Tolstov SN, Rebrov AP (2020). Altered vascular stiffness and central pulse wave parameters in climacteric women. Akusherstvo, Ginekologia i Reprodukcia.

[22] Harlow SD, Gass M, Hall JE, Lobo R, Maki P, Rebar RW, et al. STRAW 10 Collaborative Group. Executive summary of the Stages of Reproductive Aging Workshop +10: addressing the unfinished agenda of staging reproductive aging. Menopause 2012;19:387–95. 10.1097/gme.0b013e31824d8f40 PMC3340903 22343510

[23] Ageenkova OA, Purygina MA (2011). Central aortic blood pressure, augmentation index, and reflected wave transit time: reproducibility and repeatability of data obtained by oscillometry. Vasc Health Risk Manag.

[24] Kotovskaya YV, Kobalava ZD, Orlov AV (2014). Validation of the integration of technology that measures additional «vascular» indices into an ambulatory blood pressure monitoring system. Medl Dev Evid Res.

[25] Rogoza AN, Kuznetsov AA (2012). Central aortic blood pressure and augmentation index: comparison between Vasotens and SphygmoCor technology. Res Rep Clin Card.

[26] Kuznetsova TY, Korneva VA, Bryantseva EN, Barkan VS, Orlov AV, Posokhov IN (2014). The 24-hour pulse wave velocity, aortic augmentation index, and central blood pressure in normotensive volunteers. Vasc Health Risk Manag.

[27] Sarafidis PA, Loutradis C, Karpetas A, Tzanis G, Piperidou A, Koutroumpas G (2017). Ambulatory pulse wave velocity is a stronger predictor of cardiovascular events and all-cause mortality than office and ambulatory blood pressure in hemodialysis patients. Hypertension.

[28] Tolstov SN, Salov IA, Rebrov AP (2018). Structural and functional changes of blood vessels in women in early postmenopause, the possibility of correction of the revealed violations. Kardiologiia.

[29] Williams B, Lacy PS, Baschiera F, Brunel P, Düsing R (2013). Clinical trial: the ambulatory central aortic pressure (AmCAP) study blood pressure and the impact of blood pressure treatment in a randomized controlled novel description of the 24-h circadian rhythms of brachial versus central aortic. Hypertension.

[30] Kaess BM, Rong J, Larson MG, Hamburg NM, Vita JA, Cheng S (2016). Relations of central hemodynamics and aortic stiffness with left ventricular structure and function: The Framingham Heart Study. J Am Heart Assoc.

[31] Hayward CS, Knight DC, Wren BG, Kelly RP (1997). Effect of hormone replacement therapy on non-invasive cardiovascular haemodynamics. J Hypertens.

